# Efficacy of single-dose galcanezumab 240 mg on episodic or refractory chronic cluster headache: prospective, 4-week, real-world evidence from the GRASP study group

**DOI:** 10.3389/fneur.2025.1748498

**Published:** 2026-01-12

**Authors:** Michail Vikelis, Andreas A. Argyriou, Maria Chondrogianni, Aikaterini Foska, Dimitrios Rikos, Christos Tsironis, Panagiotis Soldatos, Maria Koutsokera, Emmanouil V. Dermitzakis

**Affiliations:** 1 Glyfada Headache Clinic, Glyfada, Greece; 2 Headache Outpatient Clinic, Department of Neurology, Agios Andreas General Hospital of Patras, Patras, Greece; 3 Second Department of Neurology, National and Kapodistrian University of Athens, School of Medicine, "Attikon" University Hospital, Athens, Greece; 4 Department of Neurology, 404 Military Hospital, Larisa, Greece; 5 Headache Outpatient Clinic, Department of Neurology, University Hospital of Ioannina, Ioannina, Greece; 6 Kalamata Headache Clinic, Kalamata, Greece; 7 Headache Outpatient Clinic, Department of Neurology, Thriasion General Hospital, Elefsina, Greece; 8 General Clinic, Thessaloniki, Greece

**Keywords:** chronic cluster headache, effectiveness, episodic cluster headache, galcanezumab, prevention, safety

## Abstract

**Objective:**

This prospective real-world study, conducted by the Greek Research Alliance for the Study of Headache and Pain (GRASP), aimed to evaluate the efficacy, tolerability and safety of subcutaneous galcanezumab 240 mg in reducing the frequency and clinical burden of CH attacks at week 4.

**Methods:**

The primary endpoint was the change in the mean number of daily cluster attacks from baseline to weeks 2 and 4. The co-primary endpoint was the ≥50% response rate at week 4 versus baseline. Secondary endpoints included attack duration, pain intensity (VAS 0-10), quality of life (EQ-VAS), treatment satisfaction (PGIC 1–7), and galcanezumab safety and tolerability, all assessed comparing the outcomes at weeks 2 and 4 with those at the baseline.

**Results:**

Forty-seven patients with episodic (eCH, *n* = 30) or refractory chronic cluster headache (cCH, *n* = 17) received a total dose of 240 mg of galcanezumab, consisting of two pre-filled 120 mg syringes. Mean age was 43.6 years; 78.7% were male. At week 4, mean daily attacks decreased from 2.6 to 1.2 in eCH (*p* < 0.001) and from 2.8 to 1.5 in cCH (*p* = 0.004) with significant effect sizes. A response rate >50% was observed in 63% of patients with eCH and in 65% of patients with cCH. Pain intensity (VAS) and attack duration significantly improved, alongside EQ-VAS quality of life and PGIC satisfaction scores. Adverse events during the galcanezumab treatment were mild and transient, and did not lead to discontinuations.

**Conclusion:**

A single 240 mg dose of galcanezumab significantly reduced attack frequency, pain, and duration in both patients with eCH and refractory cCH, improved quality of life and patients’ satisfaction, and was well-tolerated. These real-world findings support the use of galcanezumab mostly in eCH, while larger studies are needed to clarify its role in cCH.

## Introduction

Cluster headache (CH) is a severe primary headache disorder characterized by recurrent, unilateral, excruciating pain, typically localized to the temporal, orbital, or supraorbital regions. The pain is frequently accompanied by cranial autonomic symptoms, such as lacrimation, nasal congestion, facial sweating, and eyelid edema (1). CH affects approximately 0.1% of adults and occurs more commonly in men, with a male-to-female ratio of about 3:1. The disorder usually manifests between 20 and 50 years of age but can occur at any stage of life, including in pediatric populations (2). CH is classified into two main forms. Episodic CH (eCH), accounting for roughly 80% of cases, is characterized by cluster periods lasting weeks to months, followed by remission intervals that may extend for years. Attacks may occur several times daily, lasting 15 min to 3 h if left untreated (3). Chronic CH (cCH) involves attacks persisting for 1 year or longer without remission, or with remission periods shorter than 3 months within a year (4).

Despite increased understanding of its clinical and epidemiological characteristics, CH remains frequently misdiagnosed, leading to delayed treatment initiation (5, 6). Psychiatric comorbidities, particularly anxiety and depression, are common in patients with CH and can further impact disease burden and quality of life (1, 7).

Although several acute and preventive therapies are available, an effective and universally accepted preventive treatment remains an unmet need, reflecting the disorder’s complex pathophysiology (8). In routine practice, combinations of preventive and transitional approaches are used to achieve clinically meaningful relief. Common preventive agents include verapamil, lithium, valproic acid, topiramate, and melatonin, whereas greater occipital nerve blocks and short-term corticosteroids can have a role as transitional options. However, these treatments are nonspecific, of limited efficacy, and constrained by tolerability issues (9).

Neurovascular mechanisms involving trigeminovascular system and autonomic nervous system play a critical role in CH pathophysiology (10), therefore, targeting neuropeptides within this pathway, e.g., Calcitonin Gene related Peptide – CGRP, has emerged as a promising therapeutic strategy. Although large pivotal trials of anti-CGRP monoclonal antibodies have shown modest or no preventive efficacy, CGRP blockade still remains of scientific and clinical interest (11). Specifically, Galcanezumab, which is the only approved prophylactic medication for eCH in the U.S., but not for cCH, based on results of randomized controlled trials (12, 13), has shown overall promising results in the prophylaxis of both eCH and cCH (14). Furthermore, real-world studies corroborate its effectiveness and favorable safety profile (15–18).

Given the limited national evidence reflecting real-world outcomes in Greece, the present study, conducted by the Greek Research Alliance for the Study of Headache and Pain (GRASP), aimed to prospectively evaluate the efficacy and safety of galcanezumab 240 mg in reducing the frequency and clinical burden of CH attacks at week 4 in a real-world clinical setting.

## Patients and methods

### Study design and participants

By the present observational, prospective, multicenter, real-world study we evaluated adult patients with either episodic cluster headache (eCH) or chronic cluster headache (cCH) between September 2022 and September 2025. Enrolled patients were longitudinally assessed by headache specialists at tertiary headache centers in Greece. All participants provided informed consent prior to study entry, and the study protocol was approved by the Institutional Review Board of the principal investigator. As this study is part of the GRASP study group’s ongoing cluster headache registry, it was not registered in any public clinical trial database.

The inclusion criteria were the following: (i) a definite diagnosis of eCH or refractory cCH, according to the 2018 criteria of the International Classification of Headaches Disorders-III (19); (ii) eCH patients had to have a history of inadequate response or intolerance to three previous preventive treatments; had to be in a bout for at least a week and, according to their past history of bout duration, the expected duration of the is for at least a month; (iii) Patients with cCH had to be categorized as having refractory CH (20), according the definition of experiencing at least three severe attacks per week despite at least three consecutive trials of adequate preventive treatments; (iv) patients were galcanezumab- and other antiCGRP monoclonal antibodies-naïve; (v) patients were willing to fill in diaries and other study-related questionnaires and (vi) to be contacted for additional phone interviewing to obtain any extra information where needed. Exclusion criteria included any contraindication to galcanezumab, according to the available bibliographic evidence and standard clinical practice (21).

### Intervention

The approved dosage of galcanezumab for eCH in the United States is 300 mg, administered as three consecutive 100 mg subcutaneous injections at the onset of the cluster period and continued monthly until the end of the bout. In contrast, galcanezumab is not approved for CH prevention in the European Union, following the European Medicines Agency’s decision not to grant this indication. In Greece, only the 120 mg formulation is available. For this reason, and in accordance with national reimbursement regulations for high-cost, off-label therapies, patients in this study self-administered a single total dose of 240 mg, delivered as two prefilled 120 mg syringes (Emgality® 120 mg, Eli Lilly/Organon-Hellas) at baseline. No scheduled repeat dosing was performed during the treated cluster period.

This approach differs from several European observational cohorts, which used a 300 mg dose with the possibility of repeat administration across attack periods or subsequent cluster recurrences. The choice of a fixed 240 mg single-dose regimen in our study reflects the general practical constraints of the Greek reimbursement system, which requires prior authorization and restricts coverage to patients who have failed or not tolerated at least three first-line oral preventive therapies. Under these conditions, approval for 300 mg would have been difficult to obtain.

The follow-up duration was 4 weeks for each patient. During this period, and consistent with their prior treatment history, patients did not receive additional intermediate or preventive therapies for cluster headache. They continued using their usual abortive treatments as needed during the four-week observation period, without making any changes to their acute treatment plan.

### Outcome measures

Participants maintained a paper-based daily headache diary beginning at baseline through the final follow-up assessment at week 4. Compliance was defined as documentation of at least 80% of total diary days from baseline to week 4. The primary endpoint was the change in the number of daily CH attacks from baseline to week 2 and week 4 of study participation. Co-primary endpoint was the response rate, defined as the >50% reduction in the number of daily CH attacks, measured at week 4, as compared to mean number of CH during the last 5 days before inclusion.

Secondary endpoints included: (i) duration of CH attacks from day 1 to week 2 and week 4 of study participation; (ii) pain intensity (mean/median) of CH attacks at week 2 and at week 4 after initial treatment as measured by the Visual Analogic Rating Scale (VAS), which is an 11-point pain intensity numerical rating scale ranging from 0 = no pain to 10 = worst possible pain (22); (iii) impact of treatment on self-perceived quality of life (QOL), assessed between baseline and week 2 and week 4 using the EQ-VAS, a thermometer-like vertical scale from 0 to 100, where 0–40 indicates severely affected health, 50–70 indicates moderate impairment, and >70 indicates no impairment (23); (iv) impact of treatment on self-perceived quality of life (QOL), assessed between baseline and week 2 and week 4 using the EQ-VAS, a thermometer-like vertical scale from 0 to 100, where 0–40 indicates severely affected health, 50–70 indicates moderate impairment, and >70 indicates no impairment (24); (v) satisfaction to treatment, assessed on week 4 from baseline, measured using the self-report 7-point Patient Global Impression of Change (PGIC) questionnaire, where 1 = “no change” and 7 = “considerable improvement (25); and (vi) tolerability and safety during galcanezumab treatment were assessed recording adverse events during experienced during the treatment. Patients were encouraged to report any adverse event (AE) or serious adverse event (SAE) between days 1 and 28 post-treatment, either spontaneously or in response to direct questioning. Reported treatment-related AEs were categorized as mild, moderate, or severe and also if they could result in treatment discontinuation/decision not to continue or repeat treatment.

### Statistical analysis

Descriptive statistics were calculated according to the nature of each variable. Repeated measures ANOVA was used to evaluate changes in the primary and secondary efficacy variables from baseline to weeks 2 and 4. All tests were two-sided unless otherwise specified, with *p* < 0.05 considered statistically significant. Statistical analyses were performed using SPSS for Windows, version 27.0 (SPSS Inc., Chicago, IL, USA).

## Results

All initially enrolled patients completed their headache diaries in full; therefore, no missing data were encountered. We eventually included 47 patients with a confirmed diagnosis of either episodic (eCH: *n* = 30) or refractory chronic cluster headache (cCH: *n* = 17) who received a total dose of galcanezumab 240 mg, administered SC as two 120 mg injections for the treatment of either a CH bout in eCH or for the treatment of cCH. The majority were male (37; 78.7%), regardless of CH subtype. The mean age of participants was 43.6 ± 10.8 years (range: 21–66 years), and their mean age at first CH diagnosis was 25.3 ± 10.4 years (range: 18–40 years).

Participants had shown variable responses to previous acute or preventive CH treatments, most commonly responding to transitional therapy with steroids (oral prednisolone 1 mg/kg/day for 5 days, followed by tapering by 20 mg every 3 days). Among previously used preventive therapies, verapamil (480–900 mg/day) was the most commonly administered and the most effective. Patients had failed a median of four (range: 3–6) prophylactic treatments before initiating galcanezumab. Baseline characteristics of all study participants, both overall and stratified by CH subtype, are summarized in Table 1.

**Table 1:** Demographic and clinical characteristics of participants at baseline.

Participants	All (*n* = 47)	eCH (*n* = 30)	cCH (*n* = 17)
Gender			
Female (*n*, %)	10 (21.3%)	8 (26.7%)	2 (11.8%)
Male (*n*, %)	37 (78.7%)	22 (73.3%)	15 (88.2%)
Age ± SD (range)	43.6 ± 10.8 (21–66)	42.5 ± 10.9 (21–63)	45.5 ± 10.8 (29–66)
Age at CH diagnosis (range)	25.3 ± 10.4 (18–40)	25.4 ± 10.2 (18–36)	25.1 ± 11.2 (20–40)
Median (range) bout duration for eCH (days)	–	60 (35–180)	–
Median duration cCH	–	–	5 (3–6)
Responders to symptomatic/transitional treatments			
High Flow O_2_ (n)	20	13	7
Sumatriptan SC (n)	35	20	15
Oral prednisolone (n)	42	26	16
Number and type of previous failed preventive drugs			
Median (range)	4 (3–6)	3 (3–4)	4 (3–6)
*Calcium channel blockers*			
Verapamil (n)	38	21	17
*Mood stabilizers/anticonvulsants*			
Lithium (n)	20	4	16
Topiramate (n)	17	5	12
Gabapentin/Pregabalin (n)	27	14	13
*Peripheral/interventional approaches*			
GONB (n)	17	8	9
BoNTA (n)	2	1	1

eCH, episodic cluster headache; cCH, chronic cluster headache; SD, standard deviation; GONB, Great occipital nerve block; BoNTA, OnabotulinumtoxinA.

### Single-dose galcanezumab 240 mg for ongoing episodic cluster headache bout

Episodic CH (eCH) patients had experienced a median of 16 (range: 4–21) previous bouts prior to the ongoing bout, and the median time from the onset of the current bout to galcanezumab administration was 10 days (range: 7–23 days). The mean number of daily attacks after administration of galcanezumab 240 mg was significantly reduced from a baseline of 2.6 ± 1.0 to 1.5 ± 1.4 at week 2, and 1.2 ± 1.3 at week 4. Substantial effect sizes were evident at both assessments, with clear improvement from baseline to week 2 (z − 3.186, *p* < 0.001, effect size 0.58) and even greater improvement by week 4 (z − 3.835, *p* < 0.001, effect size 0.70). Nineteen of 30 eCH patients were classified as treatment responders, with the median monthly number of attacks at week 4 reduced by >50% (*n* = 6) or >75% (*n* = 10) compared to baseline. Galcanezumab completely terminated the cluster bout in three patients (100% response).

**Figure 1:**
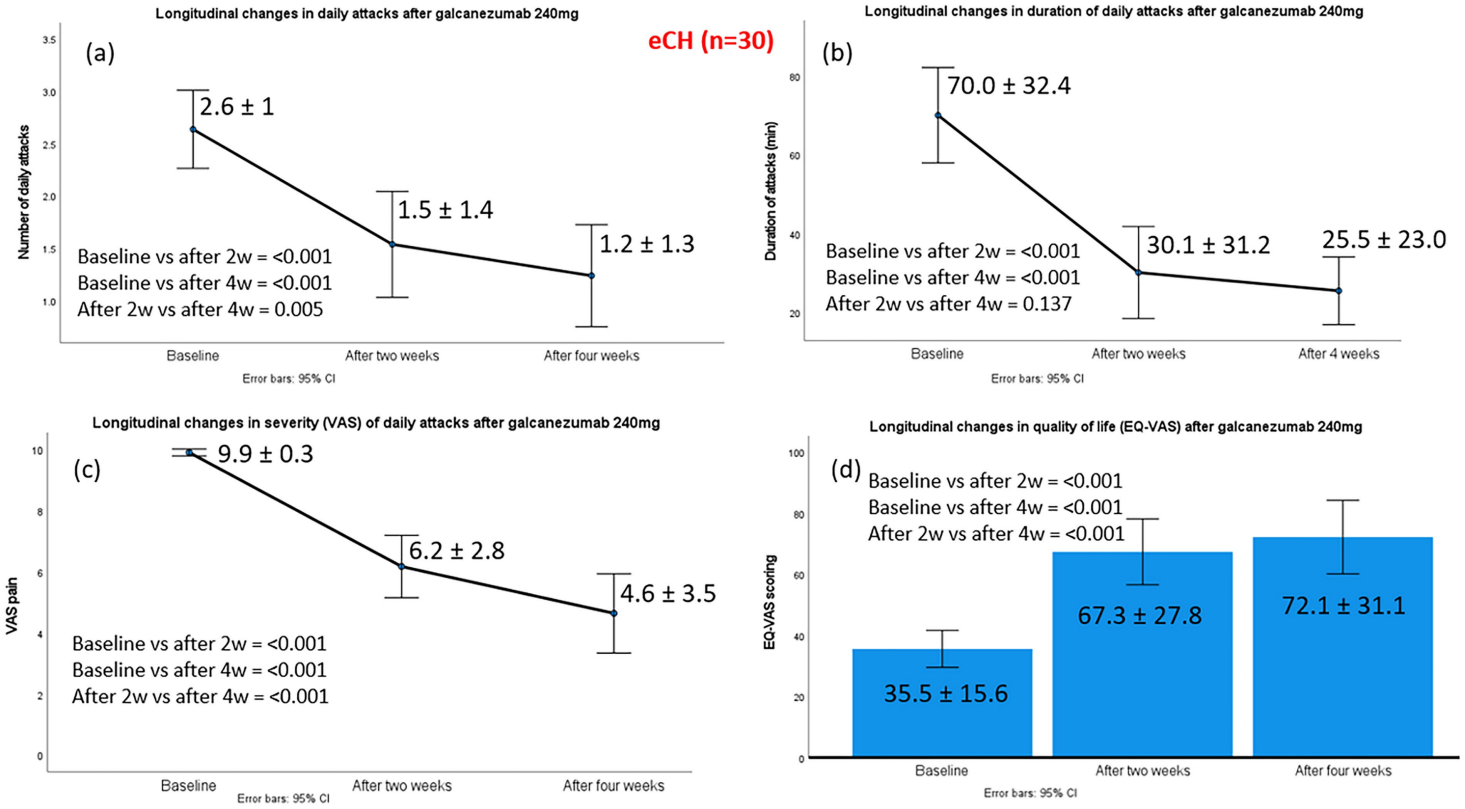
Changes in the main efficacy outcomes from baseline to week 2 and week 4, post-galcanezumab, in 30 episodic cluster headache patients. Part label **(a)** refers to changes in daily attacks; **(b)** to changes in duration of daily attacks; **(c)** to changes in severity of daily attacks and **(d)** to changes in the quality-of-life status, according to EQ-VAS.

The mean duration of CH attacks also decreased significantly, by 39.9 min at week 2 and 44.5 min at week 4. Similarly, significant improvements were observed in the pain intensity of cluster attacks, with median VAS scores declining from 10 at baseline to 7 at week 2 and 5 at week 4 (*p* < 0.001). Changes in the main efficacy outcomes from baseline to weeks 2 and 4 are illustrated in Figure 1.

Improvement in in patients’ self-reported quality of life, as assessed by EQ-VAS, was observed. Median scores increased from 30 at baseline (severely impaired health status) to 80 at week 2 and 87.5 at week 4 (indicating no impairment) following galcanezumab treatment. All 19 of the 30 treatment responders reported high levels of perceived improvement and satisfaction with galcanezumab, scoring ≥5 on the PGIC scale (6 patients scored 5, 4 scored 6, and 9 scored 7).

No systemic adverse events or new safety signals related to galcanezumab were observed. Six mild and transient adverse events were documented, including injection-site reactions such as discomfort, redness, or pruritus (*n* = 4) and flu-like symptoms (*n* = 2). No patients decided to discontinue treatment due to safety or tolerability issues.

### Single-dose galcanezumab 240 mg for refractory chronic cluster headache

The mean number of daily CH attacks in patients with cCH p after administration of galcanezumab 240 mg decreased significantly from 2.8 ± 1.4 at baseline to 1.6 ± 1.3 at week 2 and 1.5 ± 1.3 at week 4. Substantial effect sizes were observed at both time points. At week 2, the treatment produced a significant change relative to baseline (z − 2.871; p 0.004; effect size 0.69). By week 4, the magnitude of improvement increased further (z − 3.165; p 0.002; effect size 0.77).

A total of 11 of 17 patients (64.7%) were classified as treatment responders; among them, six patients (54.5%) achieved a reduction in median monthly CH attacks of ≥50% and five patients (45.5%) achieved ≥75% at week 4 compared to baseline. None of the patients with cCH experienced complete cessation of cluster attacks from administration to week 4.

**Figure 2:**
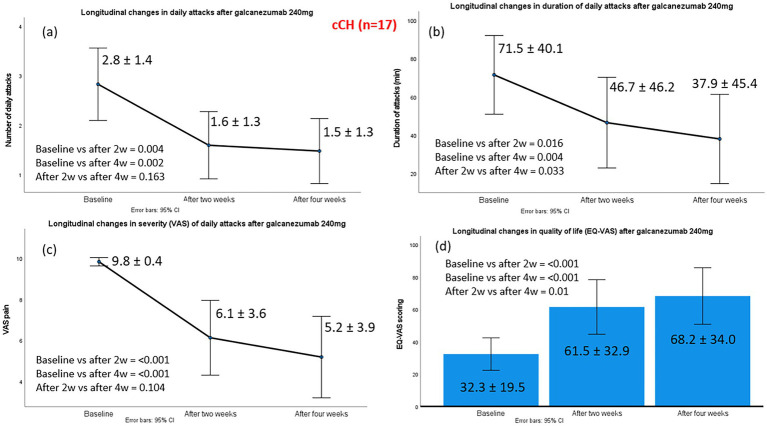
Changes in the main efficacy outcomes from baseline to week 2 and week 4, post-galcanezumab, in 17 chronic cluster headache patients. Part label (a) refers to changes in daily attacks; (b) to changes in duration of daily attacks; (c) to changes in severity of daily attacks and (d) to changes in the quality-of-life status, according to EQ-VAS.

Significant improvements were observed in the median duration of daily attacks (60 vs. 45 vs. 30 min) and in the median VAS of CH attacks (10 vs. 7 vs. 6) from baseline to weeks 2 and 4. EQ-VAS scores improved significantly, rising from a median baseline value of 30 (severely impaired health status) to 70 at weeks 2 and 4 (moderately impaired health status), reflecting a meaningful enhancement in self-perceived quality of life. Improvements in cCH patients are illustrated in Figure 2. Galcanezumab positively influenced patients’ perception of treatment, with all responders scoring ≥5 on the PGIC. Specifically, five responders scored 5, and six scored 6.

Similarly to the eCH group, there were no cases of early treatment discontinuation/decision not to continue due to safety or tolerability issues among patients with cCH, and all participants decided to continue subsequent treatment cycles as scheduled. Most reported adverse effects were mild and transient, including injection-site erythema, discomfort, redness, or pruritus (*n* = 4) and flu-like symptoms (*n* = 1).

## Discussion

In the present real-world, prospective, multicenter study, we observed that a total dose of 240 mg of galcanezumab (two single doses of 120 mg, administered on the same time) provided substantial clinical benefits for patients with both eCH and refractory cCH. Among patients with eCH, treatment with galcanezumab resulted in significant reductions in the number and duration of daily attacks, decreased pain intensity, and meaningful improvements in self-reported quality of life. Of particular note, three eCH patients experienced complete cessation of their cluster episodes, an observation that underscores the potential of galcanezumab to provide rapid control of acute bouts in selected individuals. These results are especially relevant given the often unpredictable and debilitating nature of CH attacks, where even short-term relief can dramatically improve patient functioning and well-being.

Similarly, patients with cCH demonstrated more modest, than those seen in eCH, but still clinically meaningful reductions in both the frequency and intensity of daily CH attacks, as well as shorter CH attack durations. While complete elimination of attacks was not observed in this group, a substantial proportion (64.7%) achieved a clinically relevant reduction of ≥50% in monthly attacks, reflecting the effectiveness of galcanezumab even in a population with failure to previous preventive therapies. These findings highlight the potential for galcanezumab to address an unmet need among cCH patients, many of whom have limited treatment options and often experience persistent, high-frequency attacks. Improvements in quality of life, as reflected by EQ-VAS scores, were observed in both CH patient groups, reinforcing the broader impact of effective CH prophylaxis beyond simple reductions in attack frequency.

Importantly, patients’ perceptions of treatment benefit, as measured by the Patient Global Impression of Change (PGIC), aligned closely with objective clinical outcomes. All responders reported scores ≥5, indicating moderate to considerable improvement and high satisfaction with galcanezumab treatment. This concordance between objective measures and patient-reported outcomes emphasizes the relevance of including patient perspectives (the so-called patients’ reported outcomes) when evaluating the real-world effectiveness of novel therapies. Taken together, these findings suggest that galcanezumab not only reduces attack frequency and severity but also positively impacts patients’ overall quality of life, offering a meaningful therapeutic option for individuals with both episodic and chronic forms of cluster headache. Because the abortive treatment plan remained stable throughout the study period, no meaningful impact on the reductions in attack duration or intensity could be anticipated.

Our findings are generally in agreement with most of the available real-world studies, although it should be noted that direct comparison cannot be made because of methodological differences. In a Greek study, enrolling 11 patients with refractory cluster headache (eCH *n* = 5, cCH *n* = 6), adjunctive galcanezumab (120–360 mg monthly for 3 months) was assessed for efficacy and safety. All had prior oral steroids or greater occipital nerve blocks. After treatment, weekly attacks dropped from 16.0 ± 9.4 to 1.8 ± 1.32 (*p* = 0.002), and days needing symptomatic treatment fell from 6.50 ± 3.59 to 1.8 ± 3.36 (*p* = 0.001). Two cCH patients showed no improvement. No serious adverse events were reported (22). Another study evaluated 47 patients with eCH (CH) who received ≥1 dose of galcanezumab 240 mg. Galcanezumab was started a median of 18 days after bout onset. Median time to complete cessation of weekly attacks was 17 days, with 78.8% of patients achieving ≥50% reduction by week 3 (15). In a Korean real-world study, 16 patients with eCH received two courses of galcanezumab, showing ≥50% reduction in daily headache frequency in 86% during the first episode and 64% during the second. Two patients treated pre-cluster experienced no typical attacks, suggesting potential preventive benefits (26). Moreover, 44 Brazilian patients with eCH or cCH (86.4% men; mean age 45.9 years) were treated with galcanezumab, mostly at 300 mg, achieving ≥50% headache reduction at 3 weeks in 65.9% overall and 72.4% at 300 mg (18). Finally, 21 patients with cCH, previously treated with a mean of 6.3 preventive therapies, received at least one 240 mg dose of galcanezumab. After 1 month, median attack frequency dropped from 60 to 31 per month (*p* = 0.003) and pain intensity from 10 to 8.5 (*p* = 0.007), with 47.6% achieving ≥50% reduction. At 3 months, 46.6% maintained ≥50% reduction (17).

Compared with these studies, our findings stand out in several ways. First, the use of a total dose 240 mg regimen demonstrates meaningful clinical benefits without the need for repeated administration, which may reduce treatment burden and improve adherence in real-world practice. Tellingly, our cohort received a fixed single administration of 240 mg, which reflects local availability and reimbursement constraints rather than an intentional deviation from prior European regimens. Second, our inclusion of both eCH and cCH patients allows for direct comparison of treatment effects across subtypes, showing complete cessation in select eCH patients and substantial reductions in the refractory cCH population. Third, our integration of patient-reported outcomes alongside objective clinical measures provides a more holistic evaluation of treatment impact, highlighting improvements in perceived quality of life and overall satisfaction that are not consistently reported in other studies. Other studies generally report percentage reduction ≥50% but not full cessation, except one which reports the median time to complete cessation (15). Our cCH results (64.7% ≥ 50% reduction) are broadly similar to Perez et al. study (17), though differences in baseline severity, prior therapies, and follow-up duration exist. However, it should be acknowledged that the Spanish study (17) used the 300 mg galcanezumab dosage and as such any direct comparison should be interpreted cautiously.

Although the confidence intervals for daily attack frequency and attack duration partially overlap in our cCH patients, the model-based comparisons demonstrated statistically significant differences. Overlapping intervals reflect variability within groups and do not invalidate the between-group contrast, which is assessed by the ANCOVA estimates and their associated standard errors. To address clinical relevance, we report the absolute reductions in attack burden and the magnitude of improvement observed in our cCH cohort supports that the treatment effect is not only statistically significant but also clinically relevant. Nonetheless, taken together, these differences suggest that galcanezumab can provide clinically meaningful and patient-centered benefits in both episodic and refractory chronic cluster headache populations, complementing and extending the efficacy findings of prior real-world investigations.

From a safety and tolerability perspective, galcanezumab was well tolerated in both of our eCH and cCH patients. Adverse events were generally mild, transient, and primarily limited to injection-site reactions or flu-like symptoms, with no patients discontinuing therapy due to safety concerns. These findings are consistent with previous clinical trials, reinforcing the favorable risk–benefit profile of galcanezumab also for cluster headache prevention (14).

Considering that our study is an open-label, short-term observational study without a control group, we should acknowledge several limitations including, the high likelihood of natural remission in eCH; potential placebo and regression-to-mean effects; inability to attribute observed improvements solely to galcanezumab; lack of blinding and lack of comparator group. Additional limitations of this study include the short duration of follow-up, which may limit the generalizability of the results and the ability to assess long-term efficacy and safety. Moreover, prior RCTs with galcanezumab used 300 mg for eCH, making comparison difficult. Finally, we cannot provide comparative efficacy data with other anti-CGRP Mabs, such as erenumab, fremanezumab or eptinezumab, because these medications lack formal approval to be administered in the prevention of CH and as such cannot be reimbursed in our country even off-label.

However, the strength of our study is that although it actually confirms previous real-world findings of galcanezumab efficacy in CH prevention, it adds unique contributions. Specifically, we provide single-dose effectiveness, larger sample size than most of the previous reports, inclusion of both eCH and cCH, use of patient-reported outcomes, and rare complete cessation in eCH, which are not consistently reported in other real-world studies.

In conclusion, and considering our moderate sample size, a total 240 mg dose of galcanezumab was associated with meaningful clinical benefits in eCH and also showed effectiveness in refractory cCH, including reductions in attack frequency, pain intensity, and duration, alongside improvements in quality of life and patient satisfaction at week 4. The treatment was generally well tolerated, with only mild, transient adverse events and no safety-related discontinuations. These real-world findings support galcanezumab as a useful and safe therapeutic option for managing ongoing CH episodes, particularly in eCH, and suggest it may offer benefits in cCH patients, despite more modest and variable responses. Larger, controlled studies with extended follow-up are needed to confirm these observations and better define long-term efficacy, optimal dosing, and predictors of treatment response across both eCH and cCH populations.

## Data Availability

The raw data supporting the conclusions of this article will be made available by the authors, without undue reservation.
